# Patterns of practice for adaptive and real-time radiation therapy (POP-ART RT) part II: Offline and online plan adaption for interfractional changes

**DOI:** 10.1016/j.radonc.2020.06.017

**Published:** 2020-12

**Authors:** Jenny Bertholet, Gail Anastasi, David Noble, Arjan Bel, Ruud van Leeuwen, Toon Roggen, Michael Duchateau, Sara Pilskog, Cristina Garibaldi, Nina Tilly, Rafael García-Mollá, Jorge Bonaque, Uwe Oelfke, Marianne C. Aznar, Ben Heijmen

**Affiliations:** a Joint Department of Physics, The Institute of Cancer Research and The Royal Marsden NHS Foundation Trust, United Kingdom; b Division of Medical Radiation Physics, Department of Radiation Oncology, Inselspital, Bern University Hospital, University of Bern, Switzerland; c Department of Medical Physics, Royal Surrey County Hospital, St. Luke’s Cancer Centre, Guildford, United Kingdom; d Cancer Research UK VoxTox Research Group, University of Cambridge Department of Oncology, Cambridge Biomedical Campus, Addenbrooke’s Hospital, United Kingdom; e Amsterdam UMC, Department of Radiation Oncology, The Netherlands; f Department of Radiation Oncology, Radboud University Medical Center, Nijmegen, The Netherlands; g Applied Research, Varian Medical Systems Imaging Laboratory GmbH, Dättwil, Switzerland; h MIM Software Inc., Cleveland, United States; i Department of Oncology and Medical Physics, Haukeland University Hospital, Bergen, Norway; j Department of Physics and Technology, University of Bergen, Norway; k IEO, European Institute of Oncology IRCCS, Unit of Radiation Research, Milan, Italy; l Elekta Instruments AB, Stockholm, Sweden; m Medical Radiation Physics, Department of Immunology, Genetics and Pathology, Uppsala University, Sweden; n Servicio de Radiofísica y Protección Radiológica, Consorcio Hospital General Universitario de Valencia, Spain; o Servicio de Radiofísica y Protección Radiológica, Consorcio Hospitalario Provincial de Castellón, Castelló de la Plana, Spain; p Division of Cancer Sciences, Faculty of Biology, Medicine and Health, The University of Manchester, The Christie NHS Foundation Trust, United Kingdom; q Nuffield Department of Population Health, University of Oxford, United Kingdom; r Erasmus MC Cancer Institute, Department of Radiation Oncology, Rotterdam, The Netherlands

**Keywords:** Adaptive radiotherapy, Plan library, Plan of the day, Image-guided radiotherapy (IGRT), MR-guided radiotherapy, Interfractional motion

## Abstract

**Purpose:**

The POP-ART RT study aims to determine to what extent and how intrafractional real-time respiratory motion management (RRMM), and plan adaptation for interfractional anatomical changes (ART) are used in clinical practice and to understand barriers to implementation. Here we report on part II: ART using more than one plan per target per treatment course.

**Materials and methods:**

A questionnaire on the current practice of ART, wishes for expansion or implementation, and barriers to implementation was distributed worldwide. Four types of ART were discriminated: daily online replanning, online plan library, protocolled offline replanning (all three based on a protocol), and ad-hoc offline replanning.

**Results:**

The questionnaire was completed by 177 centres from 40 countries. ART was used by 61% of respondents (31% with protocol) for a median (range) of 3 (1–8) tumour sites. CBCT/MVCT was the main imaging modality except for online daily replanning (11 users) where 10 users used MR. Two thirds of respondents wished to implement ART for a new tumour site; 40% of these had plans to do it in the next 2 years. Human/material resources and technical limitations were the main barriers to further use and implementation.

**Conclusions:**

ART was used for a broad range of tumour sites, mainly with ad-hoc offline replanning and for a median of 3 tumour sites. There was a large interest in implementing ART for more tumour sites, mainly limited by human/material resources and technical limitations. Daily online replanning was primarily performed on MR-linacs.

## Highlights

• The patterns of practice for adaptive radiotherapy were evaluated for 177 centres.
• Over half performed ad-hoc adaption but less than a third used specific protocols.
• CBCT was the main imaging modality in general but MR was used for daily replanning.
• 2/3 centres wished to implement ART; 40% of them had plans to do it within 2 years.
• The main barriers were human/material resources and technical limitations.

Radiation therapy (RT) is usually delivered over several fractions using a treatment plan optimised on a CT-scan obtained days or even weeks prior to treatment start. However, several tumour sites present important anatomical variations during the course of treatment, which can happen on various time-scales from seconds to weeks [1]. Population-based margins [2], used to increase the probability of target coverage, may result in large irradiated volumes, potentially leading to prohibitive toxicity risks, and/or hampering tumour dose escalation. Image-guided radiotherapy (IGRT) has enabled considerable margin reduction by improving set-up accuracy [3]. Yet, anatomical changes caused by weight loss, tumour regression, variations in organ filling, or other target and organ shape changes cannot be solely addressed with translational and/or rotational set-up corrections [1]. Adaptive RT (ART), using more than one treatment plan per target per treatment course aims at counteracting the negative dosimetric impact of these changes, potentially improving target coverage and/or organ at risk (OAR) sparing with respect to the original plan [4]. Depending on the approach and tumour site, the need for a change in treatment plan is derived from offline or in-room (online) imaging [5], [6].

Offline adaption is suitable for systematic or slow progressive changes (e.g. tumour regression, weight loss) [6]. The decision to adapt can be taken ad-hoc by the treatment team based on an observed deviation in anatomy (on imaging or visible physical alterations), or following a protocol with predefined action levels and/or surveillance scans [4], [7], [8], [9], [10]. Online adaption using a plan library is well suited for tumours with predictable, potentially large and frequent interfractional anatomical variations while intrafraction changes remain comparatively small. Examples are bladder [11], [12], cervix [13], [14] or rectum [15], [16], [17] where different bladder or rectal fillings can be anticipated and a library of plans covering several scenarios are made available for treatment. Online daily replanning can address any type of anatomical changes but is the most resource-demanding approach and as such, its clinical implementation has only been demonstrated in few treatment sites and institutions so far [18], [19], [20], [21], [22].

Challenges to the clinical use of ART include the added workload [4], longer daily treatment time [5], limited image quality [23], RTT training [24], [25], uncertainty in dose accumulation [26], and software or workflow implementation [5], [27].

Despite these challenges, there is growing evidence that ART can provide a favourable dosimetric and clinical outcome compared to standard IGRT potentially allowing for safe margin reduction [8], [11], [20], [23], [28]. The patterns of practice for adaptive and real-time radiation therapy (POP-ART RT) survey was developed to determine to which extent and how real-time RT and ART are used in clinical practice for external beam photon RT, and to understand the barriers to implementation or further use to help promote the safe and effective use of these methods as a standard of care. The present paper addresses the second part: ART for interfractional anatomical changes^1^ using multiple plans per tumour and treatment course. Intrafractional anatomical changes caused by respiration can be mitigated by real-time respiratory motion management (RRMM) [29], which is the topic of an accompanying paper [30].

## Materials and methods

The web-based questionnaire, developed during the 2nd ESTRO physics workshop and further described in [30] and the supplementary materials, contained 16 questions covering ART. Data were collected between February and July 2019. The questionnaire was mainly addressed to clinical physicists but surveyed institutional practice. Centres that did not perform ART (yet) were encouraged to respond nonetheless and fill the wish-list and barriers questions.

Similar subgroup analysis to that of part I [30] was performed based on type of institutions (academic, public, private), socio-economic status [31], [32] (low, middle, high-income) and patient volume (<1000, 1000–2000, >2000 patients per year).

### Patterns of practice for ART

Four ART strategies were considered (question (Q) 1, page (P) 18):

1. offline ad-hoc (e.g. occasional detection of tumour shrinkage, weight loss)
2. offline protocol using either:
  a. pre-defined action levels based on in-room imaging (e.g. geometric deviations above a certain threshold on CBCT, observed by RTT) with referral of the decision to adapt to the clinician/physicist for subsequent fractions
  b. using scheduled surveillance scans (e.g. at given fraction numbers) and the decision to adapt is taken either by the clinician or based on objective measures similar to a.
3. online using a plan library
4. online using daily replanning.

Respondents using offline ART (1 or 2 above), were asked which percentage of the patients were getting more than one plan per tumour and course (Q2, P19, not applicable for online approaches).

Respondents using ART (“users” hereafter) were asked for each tumour site:

– what type of imaging was used to guide ART and the reasons for adaption (Q3/4 P19/20)
– what type of software was used for the ART procedure (Q5/6, P20/21)
– what additional quality assurance (QA) was performed on the adapted plan (Q7, P22)
– how was adaption documented (Q8, P23).

### Wish-lists and barriers

Similar to part I [30], users were asked if they wished to increase their use of ART or modify their technique in the next two years and for which tumour site in priority (P24) and to rank barriers in order of importance (barriers not considered relevant were not ranked) (P25).

All respondents (users and non-users) were asked if they wished to implement ART for any new tumour site and which one(s) in priority (P27). Barriers to implementation were also ranked (P28).

## Results

The ART questions were completed by 177 institutions from 40 countries (Table A.1). Sixty-one percent (108/177) of respondents were users of ART for a median (range) of 3 (1–8) tumour sites (Fig. A.1). However only 31% were using online or offline protocols for at least one tumour site (maximum 7) (Table 1, Fig. A.1). The largest group treated with a protocol was bladder (16% of respondents), dominated by the plan library strategy (15%). Offline ad-hoc adaption was performed by half the respondents, with head and neck and lung cancer being the largest groups across all subgroups of respondents (Table 1, Table A.2).

**Table 1:** Percentages of respondents (*N* = 177) that apply certain types of ART for specific tumour sites or overall.

Type of adaption	Online plan library	Online daily replanning	Offline protocol	Online or offline protocols	Offline ad-hoc	Any ART
Bladder	15%	0	1%	16%	11%	**27%**
Cervix	6%	2%	5%	13%	19%	**32%**
Rectum	1%	2%	2%	5%	13%	**18%**
Prostate^1^	<1%	3%	6%	10%	18%	**28%**
Head and Neck	0	0	10%	10%	45%	**55%**
Lung	0	0	8%	8%	28%	**36%**
Breast^1^	0	0	<1%	<1%	5%	**6%**
**Any site**	**17%**	**6%**	**15%**	**31%**	**50%**	**61%**

1 Unspecified type of adaption for one user each.

In addition to the tumour sites explicitly mentioned in the questionnaire and indicated in Table 1, four respondents used ART for sarcoma (offline ad-hoc), two for anal canal (offline, one protocolled and one ad-hoc), two for oesophagus (one daily replanning on MR-linac, one offline protocolled), two for lymphoma (one ad-hoc, one not specified), one for oligometastatic lymph nodes (plan library), one for cranial SRS (offline ad-hoc) and one respondent for liver, pancreas and abdomino-pelvic metastases (online daily replanning on MR-linac).

The use of online or offline protocols was dominated by academic centres where 48% of the respondents used such methods, while this was reduced to 24% and 28% for private and public centres respectively (Table A.2). Private centres also differed in the most common group for protocolled ART – cervix and head and neck – instead of bladder. Only 6% of respondents applied online replanning for at least one treatment site (Table 1), with the highest percentage observed for academic centres, and no application in middle-income countries (Fig. A.2).

**Fig. 1:**
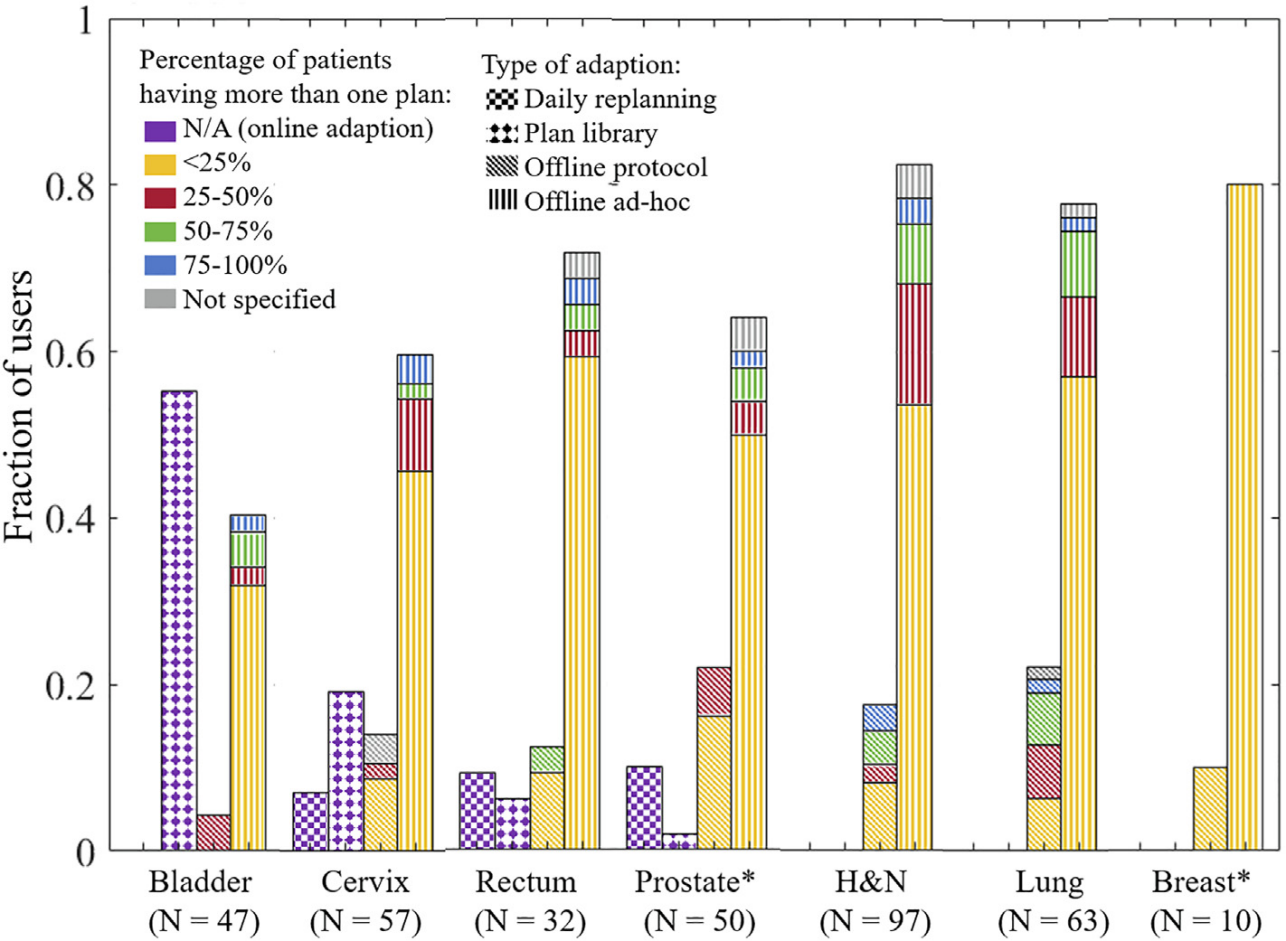
For the various tumour sites, fractions (bar heights) of users applying the defined four types of ART (bar pattern). Colours show percentage of patients having more than one plan for the offline approaches. For breast and prostate, one institution did not specify the type of ART.

For selected tumour sites, the fractions of users applying specific ART strategies are shown in Fig. 1 with colour-coding for the percentages of patients receiving more than one plan for the offline approaches (not applicable for online ART). For lung and head and neck cancer, 50% or more of the patients were replanned by less than 20% of the users of ad-hoc adaption. This increased to 35% by the users of offline protocols.

**Fig. 2:**
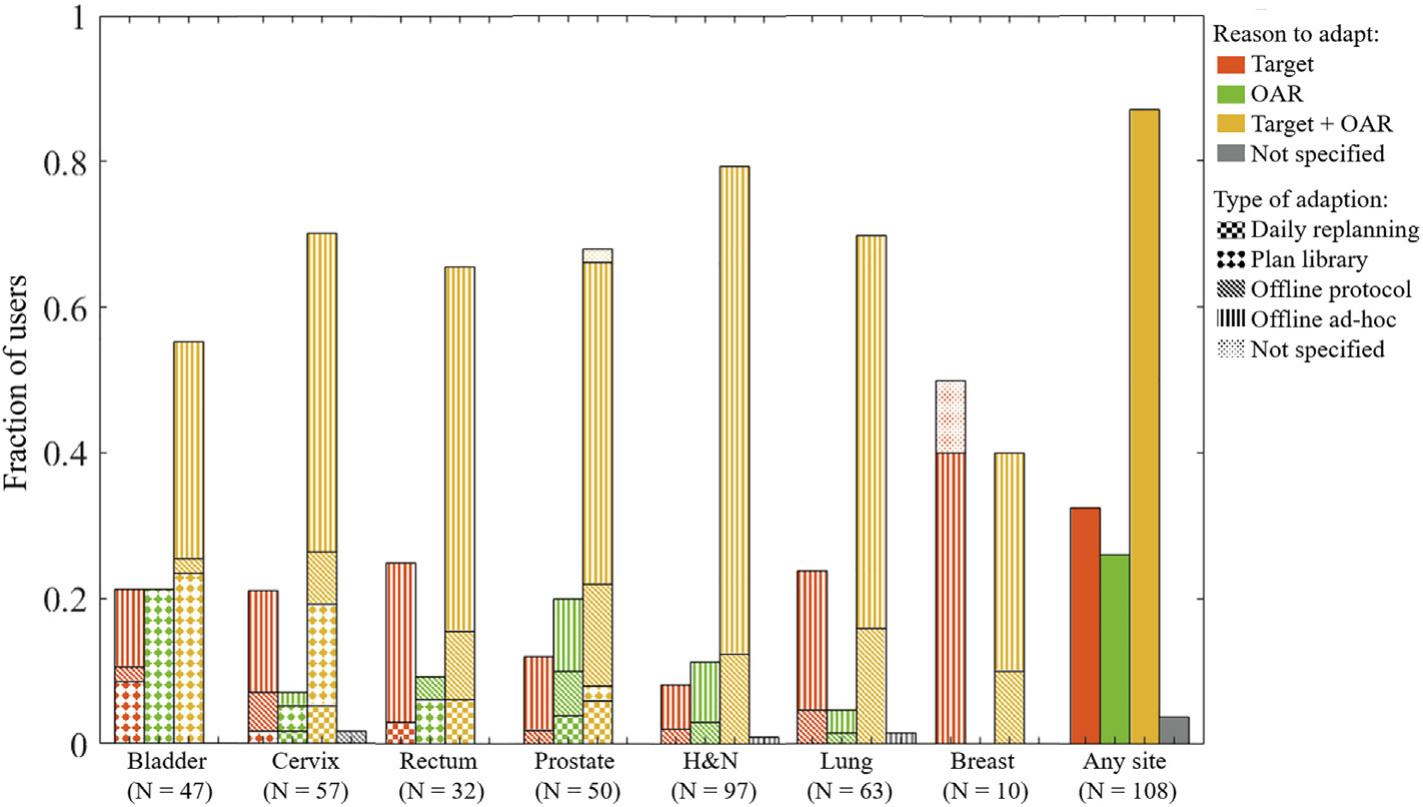
For the various tumour sites, fractions of users that apply ART to recover target dose and/or to improve OAR sparing. Bar patterns indicate which type of ART is performed for site-specific graphs. Note that due to the mix of technique for different tumour sites, the bars for “any” do not have a pattern indicating technique.

**Fig. 3:**
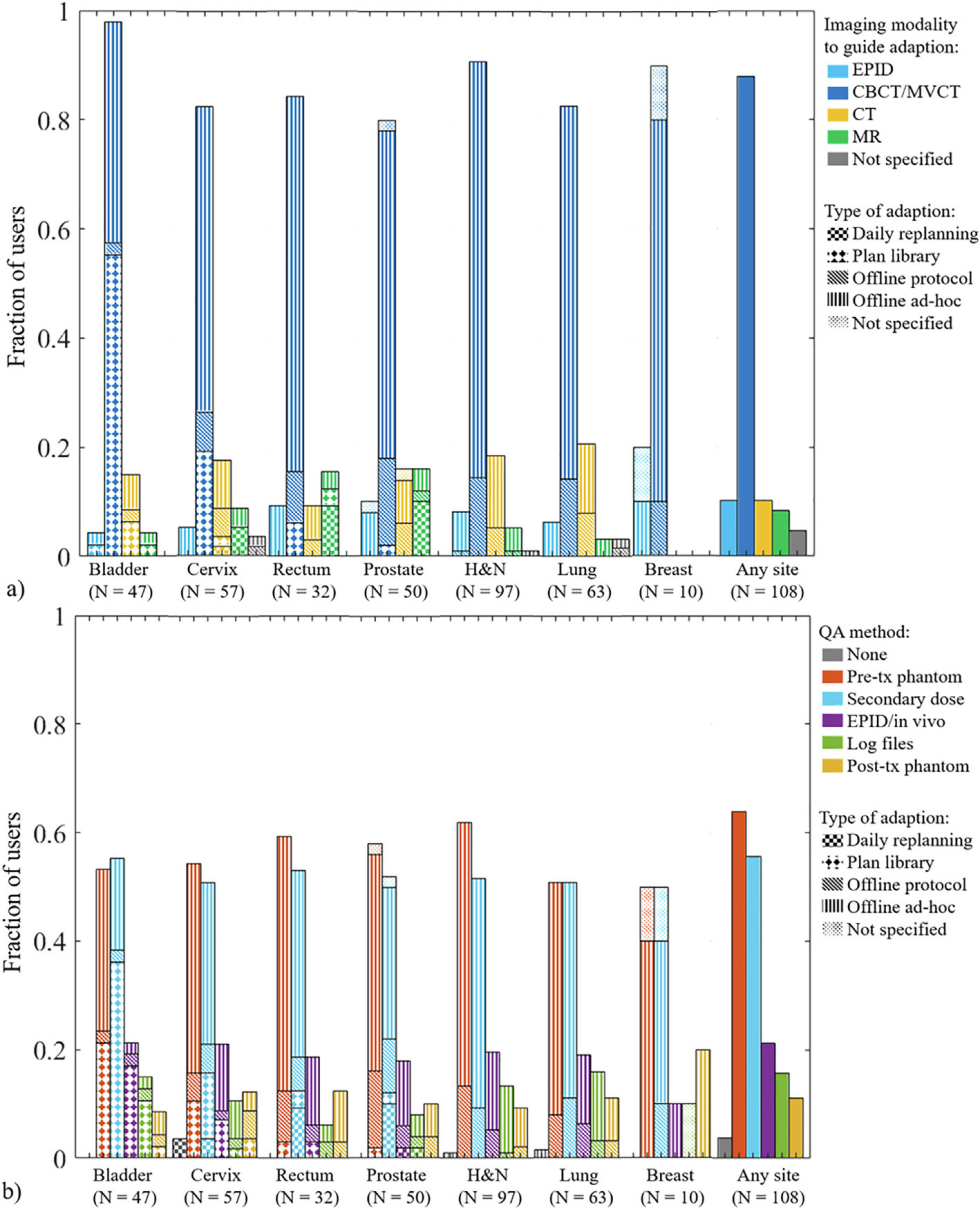
(a) For the various tumour sites, fractions of users that use given imaging modalities to guide adaption (more than one response possible) (b) fractions of users that apply given QA methods (more than on response possible). Bar patterns point at the four defined types of ART. Not that due to the mix of technique for different tumour sites, the bars for “any” do not have a pattern indicating technique.

Most adaptions were aimed at improving both target coverage and OAR sparing (Fig. 2, Fig. A.3). The main imaging modality for ART was CBCT/MVCT (>80%), while EPID was used by up to 20% of the users for offline adaption (Fig. 3a). A substantial proportion of users reported using CT or MR in combination with other imaging techniques. The use of MR was highest in academic centres, while the use of CT was highest in middle-income countries (Fig. A.4a). In addition, three users reported “poor mask fitting” as a trigger for ad-hoc adaption in head and neck cancer. Of the eleven users of online daily replanning, one used CT (cervix) while all others used MR.

Pre-treatment phantom measurements and secondary dose calculations were the most common forms of QA (Fig. 3b). Four users reported doing no QA on the adapted plan for at least one adaptive site. For users from middle-income countries, QA was mostly performed with pre-treatment phantom measurements and no user used log files (Fig. A.4b).

Although 92% of users used commercial software for the adaption procedure, 19% used in-house software alone or in combination with commercial software. None used open-source software. The lack of functionalities in commercial software was the main reason for using in-house software. Half of the users using only in-house software for head and neck and lung adaption reported the cost as the reason not to use commercial software.

Plan adaption was documented in the record-and-verify system for a majority of users. Four used only spreadsheet to record adaption while four used spreadsheet and record-and-verify. Two users did not document adaption while one used different methods: record-and-verify on the MR-linac, spreadsheet or no reporting for offline or plan library adaption.

**Fig. 4:**
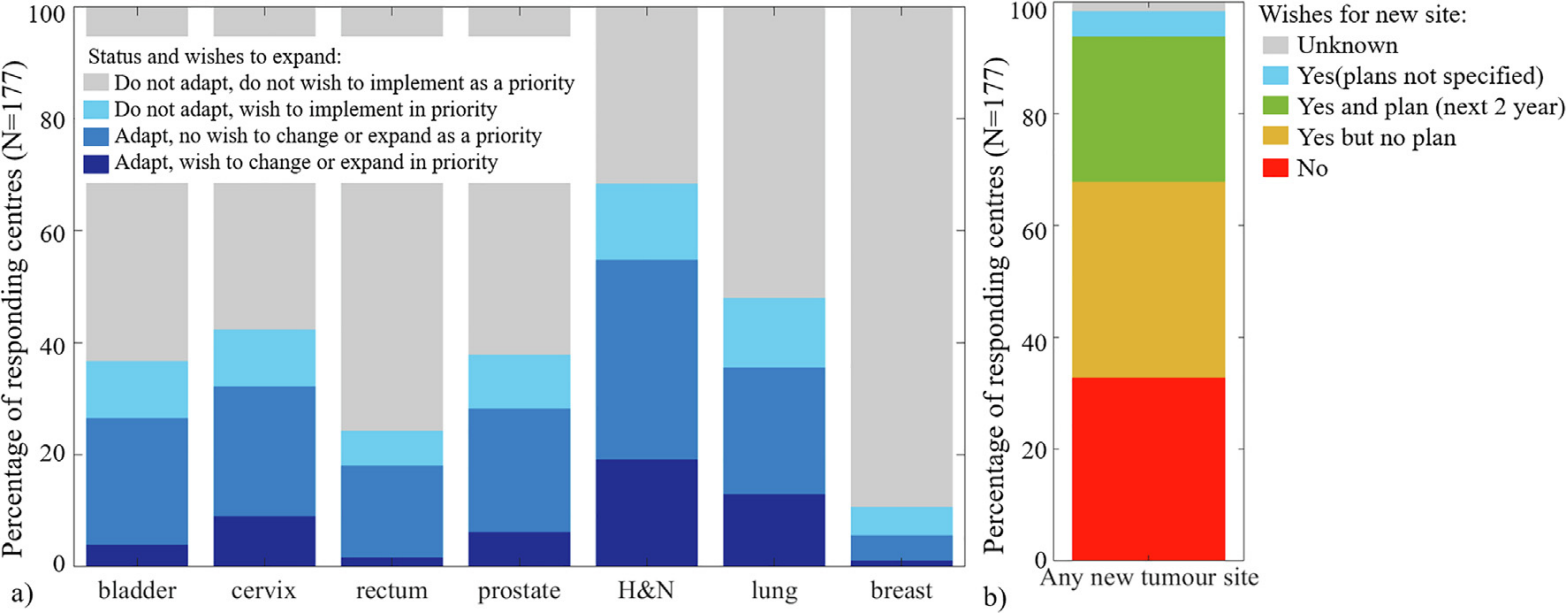
(a) For the various tumour sites, fractions of ART users that wish to change technique or increase the rate of adaption (dark blue) or not (medium blue) as a priority. Respondents not applying ART (non-users) but wishing to implement it to this site in priority (light blue) or not (grey). (b) Overall fractions of respondents (current users and non-users) wishing to implement ART for any new tumour site (blue, green and yellow) or not (red). (For interpretation of the references to colour in this figure legend, the reader is referred to the web version of this article.)

Nineteen and 13% of respondents wished to increase their use of ART or change their technique for head and neck and lung cancer respectively (representing over 35% of the users for both). In addition, 14 and 12% of respondents were not applying ART but wished to implement it in priority for head and neck and lung cancer respectively (Fig. 4a). Overall, two thirds of all 177 respondents wished to implement ART for a least one new tumour site; 40% of these had plans to do it in the next 2 years (Fig. 4b). In addition to the selected tumour sites, priority for implementing ART was given to liver (five respondents), pancreas (seven respondents) and oesophagus (two respondents) while 12 did not specify a tumour site.

**Fig. 5:**
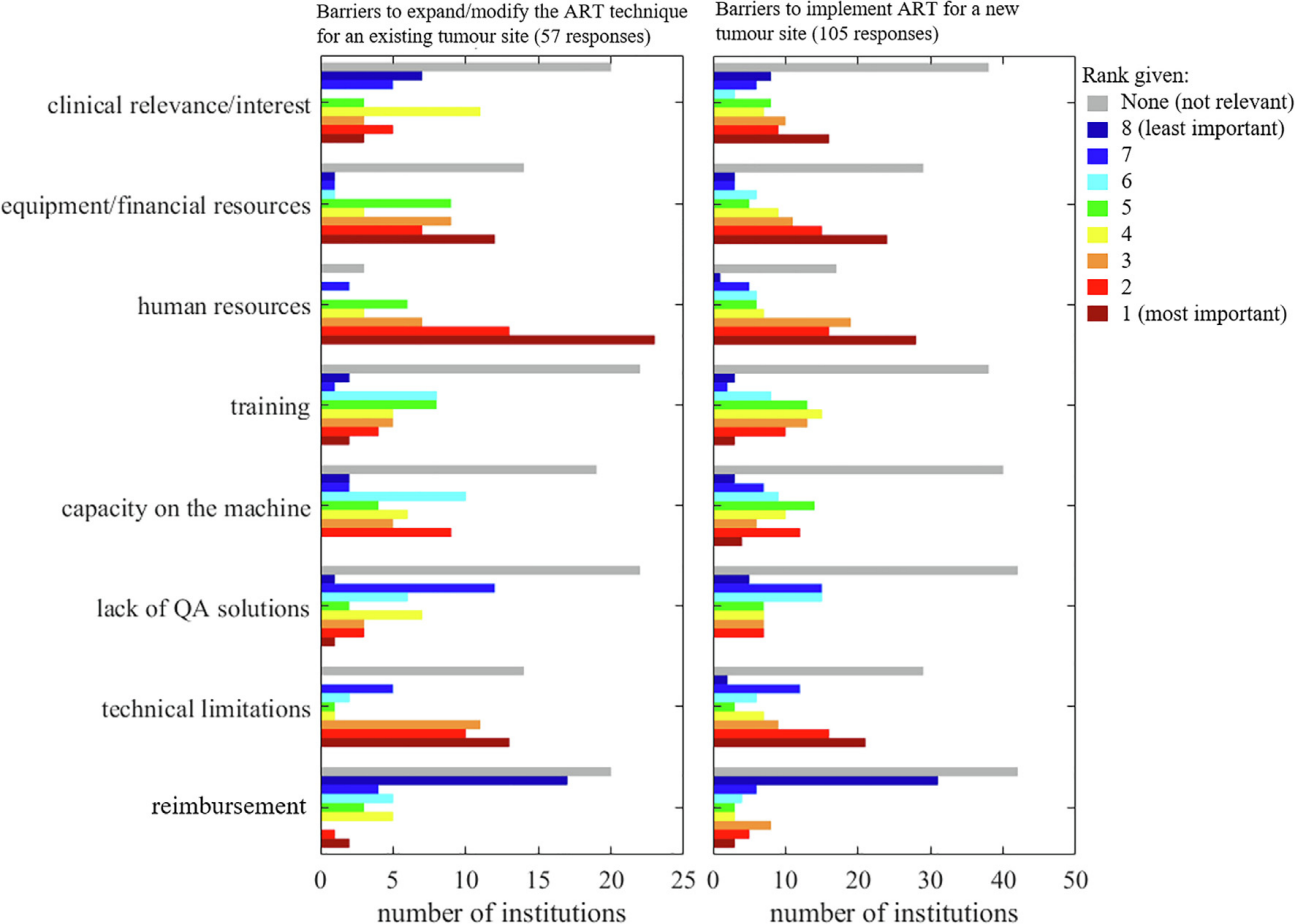
Histogram of ranks given to the barriers to further use for an existing ART tumour site (left) or implementation for a new ART tumour site (right). A lower rank (towards red) indicates high importance while a higher rank (towards blue) indicates lower importance. The grey bars indicate the number of institutions that considered the barrier “not relevant”.

Fifty-seven users ranked the barriers to expand/modify their ART technique for an existing tumour site. The main barrier was *human resources*, ranked first or second by 36 users and considered “not relevant” by only three users. *Equipment/financial resources* and *Technical limitations* were also considered highly important by a majority of users while *Reimbursement* was considered “not relevant” by 20 users and of lowest importance by 17 (Fig. 5).

One-hundred-and-five respondents (users and non-users) ranked the barriers to implementing ART for new tumour sites. *Human resources* remained the main barrier with *Equipment/financial resources*, *Clinical relevance/interest* and *Technical limitations* were also highly ranked (majority of ranks 1–3). *Reimbursement* remained lowly ranked.

Barriers entered as *other* and comments on the barriers included the “lack of interest from clinicians” or “the department” (three respondents), “approval from the authorities” (one respondent) or “insurance companies” (one respondent), “lack of clear reimbursement policy” (one respondent), “small patient volume” (one respondent), and “lack of proactive adaptive protocol” (one respondent).

The ranking of barriers did not differ substantially from the overall ranking when analysed by type of institution or socio-economic status although the number of responses was occasionally very small (Fig. A.5).

The RRMM questions were completed by 200 institutions and we encourage the reader to see the accompanying paper for details [30]. All 177 respondents for ART had also completed the RRMM questions. Out of these 177 respondents, 20% were only RRMM users, 13% only ART users and 48% were both ART and RRMM users while 19% were non-users for any treatment site or technique. Lung was a common treatment site for both parts where 29% of the respondents were only RRMM users, 19% only ART users and 17% used both RRMM and ART. There was a high interest to increase the use of/implement both ART and RRMM for lung.

## Discussion

This study reports on the use of ART in 177 RT centres from 40 countries and is, to our knowledge, the first worldwide survey on the patterns of practice for ART. Sixty-one percent of the respondents used ART for a median of 3 tumour sites (Fig. A.1). While offline ad-hoc ART was the dominant strategy, more advanced forms of ART using online or offline protocols remained relatively rare(Table 1, Fig. A.1).

Head and neck and lung were the most common sites treated with ART. RRMM was also commonly used for lung cancer [30] however, mostly for SBRT. For small mobile tumours treated with SRBT, the margin reduction -and hence lower lung dose- enabled by RRMM may be clinically beneficial [33]. ART is more commonly used for locally advanced lung cancer where atelectasis is one of the main reasons for adaption [7]. Although both ART and RRMM can be used for the same patients [33], respondents using both RRMM and ART for lung cancer may use it on different patients.

Although not technically demanding, the use of offline protocols was limited, but most prevalent in head and neck and lung cancer (10% and 8% of respondents respectively) (Table 1). Offline protocols resulted in proportionally more replanning than the ad-hoc approach (Fig. 1) indicating that ad-hoc adaption may not suffice to identify all the cases that would benefit from replanning. Conversely, it may indicate that certain protocols resulted in over-use of replanning. Certain offline ART protocols use action levels based on the correlation between observable geometric changes in images and the dosimetric benefit of adaption [7], [10]. Favourable clinical outcomes have been reported with these approaches [8], [10], [23]. However, highly sensitive action levels may result in frequent adaption with little clinical gain at the cost of a high stress on human resources. Note also that some users, only rarely adapting for exceptionally large changes, may have answered they performed ad-hoc adaption for <25% of the patients while others considered this to be anecdotal and indicated not adapting for these sites. The rate of ad-hoc adaption for <25% of the patients must then be interpreted with caution.

Regarding online protocols, 17% of respondents used a plan library approach while only 6% applied daily replanning. It is unlikely that in these centres all patients within one treatment site were treated with online adaption, but this was not covered in the questionnaire.

The imaging modality used for ART was mainly CBCT/MVCT for online plan library and offline approaches (Fig. 3a) but up to 20% of the users reported using CT and/or MR imaging as well. Although every effort was made to clearly phrase the question, it remains unclear if CT/MR was used to take the decision to adapt (in-room imaging or scheduled surveillance scans) or if a CT/MR was acquired to produce the new plan once the decision to adapt had already been taken based on other criteria. It was clear that good image quality and high soft-tissue contrast were needed for online daily replanning since 10 users used MR imaging and one used CT (probably on-rail in-room CT). Three users of MR-linac for ART also used it for RRMM (gating). One used MR-linac only for gating and six used it for ART only, which can be explained by the fact that at the time of the survey, only one of the two available MR-linac platforms had RRMM capability.

There was a pronounced interest to change technique or increase the use of ART for head and neck and lung cancer (Fig. 4a). The main barriers to do so were human/material resources and technical limitations (Fig. 5). ART for head and neck and lung cancer was only performed offline (ad-hoc or with protocol) which is well suited for systematic or slow progressive changes but puts a high demand on human resources. Lung was also a common priority in the wishes to expand/implement RRMM [30] which highlights the high variability in lung anatomy both on the intra- and interfractional time-scale. These sites are clinically challenging due to poor outcome (lung) or side effects with a high impact on quality of life (head and neck), which indicates that the RT community believes in the potential of higher targeting accuracy to improve outcome.

Two thirds of respondents wished to implement ART for a new tumour site and 40% of these had plans to do so in the next 2 years (Fig. 4b). While human/material resources and technical limitations remained important barriers, the lack of clinical interest/relevance was also highly ranked indicating the need for clinical evidence of the potential benefit of ART. It should be acknowledged that the wishes and barrier ranking could represent the personal assessment of the respondent rather than the consensus opinion of the centre.

Human/material resources were the highest ranked barriers for both RRMM and ART [30]. Only techniques feasible with conventional treatment platforms were used by more than 50% of respondents (gating with breathing surrogate and offline replanning). The overall relatively low importance given to reimbursement suggests that RRMM and ART would be used more extensively, were they available on standard equipment with a minimum increase in needed resources. Documented issues for ART such as uncertainties in dose accumulation [26] and target volume adaption in case of tumour shrinkage [34], [35] were not mentioned explicitely.

The percentage of ART users was larger among academic institutions with larger patient volumes (Tables A.1 and A.2), possibly because human/material resources can potentially be (re-)allocated more efficiently than in smaller centres. Patient selection is important to adequately use these resources [10]. However, to address these barriers more generally, automation for segmentation and treatment plan optimization are needed to alleviate the planning workload [36], [37], [38], [39]. In addition, pre-treatment phantom measurement should be replaced with other, less resources-intensive and more easily automated, QA methods [5], [40], [41]. Online daily replanning was mostly reported to be performed on MR-linacs which are still a scarce resource requiring longer treatment slots and enhanced availability of clinicians and physicists at the unit than non-adaptive workflows, therefore putting considerable stress on human/material resources [20], [42]. Research in CBCT image quality [43] and dose calculation [44], [45], needed for online daily replanning on conventional equipment, is promising. But ultimately, clinical use relies on the commercial availability of such methods.

This study presents the patterns of practice at the time of data collection in a fast-moving field. Respondents could mention their plans for expansion at two years; nevertheless there would be an interest in evaluating the changes in practice in the medium-term. In particular, a platform dedicated to daily re-planning using iteratively reconstructed CBCT [46] has been introduced shortly after the data collection period and may change practice in the near-future. MR-linac systems are also likely to be more widespread in some years.

Centres doing ART or having an interest in the technique may have been more likely to answer while other possible participation bias included accessibility to the survey (on the internet and only in English) [30]. The true proportion of users may be lower than 61% [28]. Nonetheless, with 108 users, this survey gives an interesting insight in how ART is being performed currently, as well as the wishes and barriers to expansion. In addition, with 69 non-users, the survey provides useful information on barriers to implementation.

The participation bias may have been particularly important for centres from middle-income countries. With only 17 respondents, it is difficult to draw conclusions based on socio-economic status. The availability of RT equipment and staffing was reported to be related to socio-economic status in Europe [47], [48]. The human/material resources needed for daily replanning or certain RRMM techniques [30] are therefore expected to be scarcer in middle-income countries which may explain why no centre there used daily replanning or tracking. The percentage of ART users was nevertheless as high as in high-income countries, including for plan library and offline protocols. In a survey of Indian centres attending a national educational activity on ART, even higher rates of offline ART (92% for head and neck, 52% for lung and 44% for pelvis) were reported with the lack of equipment, training and tools/management support as main barriers [49].

Although the ESTRO-HERO study concluded that staffing levels in Europe are equal to or higher than the “Radiation Therapy for Cancer: Quantification of Radiation Therapy Infrastructure and Staffing Needs” (QUARTS) recommendations, it also highlights the variations among countries and acknowledges that human resources needs have increased with the increased complexity of modern RT techniques of which RRMM and ART are good examples [50], [51].

In conclusion, ART was used for a broad range of tumour sites, mainly with ad-hoc offline replanning and for a median of 3 tumour sites per user. There was a pronounced interest in implementing ART for more tumour sites, mainly limited by human/material resources and technical limitations. More streamlined workflows allowing for reduced treatment and QA time and staff, as well as high-quality soft-tissue in-room imaging (especially for daily replanning) will be key to a wider adoption of ART.

To further promote safe and effective use of both ART and RRMM and to reduce the strain on human/material resources, we recommend that users, future users and vendors work together towards efficient solutions and workflows available for use on conventional equipment. Further, consensus on best practice is needed for the establishment of clear, broadly accepted guidelines. This could also contribute to development of solid and consistent reimbursement practices.

## Conflict of interests

Jenny Bertholet and Uwe Oelfke declare that the ICR is part of the Elekta MR-linac Research consortium.

David Noble declares that he performed consultancy work for Microsoft research during the present study. The consultancy work was however not related to the present study.

Toon Roggen declares that he is an employee of Varian Medical Systems.

Michael Duchateau declares that he is an employee of MIM Software Inc.

Nina Tilly declares that she is an employee of Elekta Intruments AB.

Other co-authors have no conflict of interest to declare in relation to the present work.

## Funding

Jenny Bertholet acknowledges funding from the Stand Up to Cancer campaign for 10.13039/501100000289Cancer Research UK (C33589/A19727 and C33589/A19908) and the CRUK ART-NET Network Accelerator Award (A21993) as well as NHS funding to the 10.13039/501100000272NIHR Biomedical Research Centre at The Royal Marsden and 10.13039/501100000027The Institute of Cancer Research.

Gail Anastasi acknowledges funding from the UK National Institute for Health Research (NIHR), (Doctoral Research Fellowship). The views expressed are those of the author(s) and not necessarily those of the NHS, the NIHR or the Department of Health and Social Care.

Marianne Aznar acknowledges support from Cancer Research UK [grant no C8225/A21133] and of the 10.13039/100014653NIHR Manchester Biomedical Research Centre.

## Appendix A Supplementary data

Supplementary Fig. A.1 Histogram of the number of tumour sites treated with ART per users. Half of the users do not use any type of protocolled ART (yellow bars). The median number of tumour sites treated with ART was 3. Half of these users used protocols.

Supplementary Fig. A.2 Fractions of users that apply the defined types of ART. Data are presented for all users together (last column) and for subgroups defined by type of institution (private, public, academic) and income (middle or high-income).

Supplementary Fig. A.3 Fractions of users that use ART to recover target dose (red), to improve OAR sparing (green), to improve both target coverage and OAR sparing (yellow) or for unknown reason (grey) per type of institution and economic status. The results for the overall group are shown to the right.

Supplementary Fig. A.4 a) Fractions of users that use given imaging modalities to guide the adaption (more than one response possible) per type of institution and economic status. b) Fractions of users that apply given QA methods (more than one response possible) per type of institution and economic status.

Supplementary Fig. A.5 Histogram of ranks given to the barriers to further use or new implementation of ART by type of institution and economic status.

Supplementary data: web-based questionnaire

## Footnotes

1 Adaption to biological changes are considered beyond the scope of this study.
