# Supplementary figures and images for: Patterns of practice for adaptive and real-time radiation therapy (POP-ART RT) part II: Offline and online plan adaption for interfractional changes
Source: Radiother Oncol. 2020 Dec;153:88–96. doi: 10.1016/j.radonc.2020.06.017 (PMC7758781; doi:10.1016/j.radonc.2020.06.017)

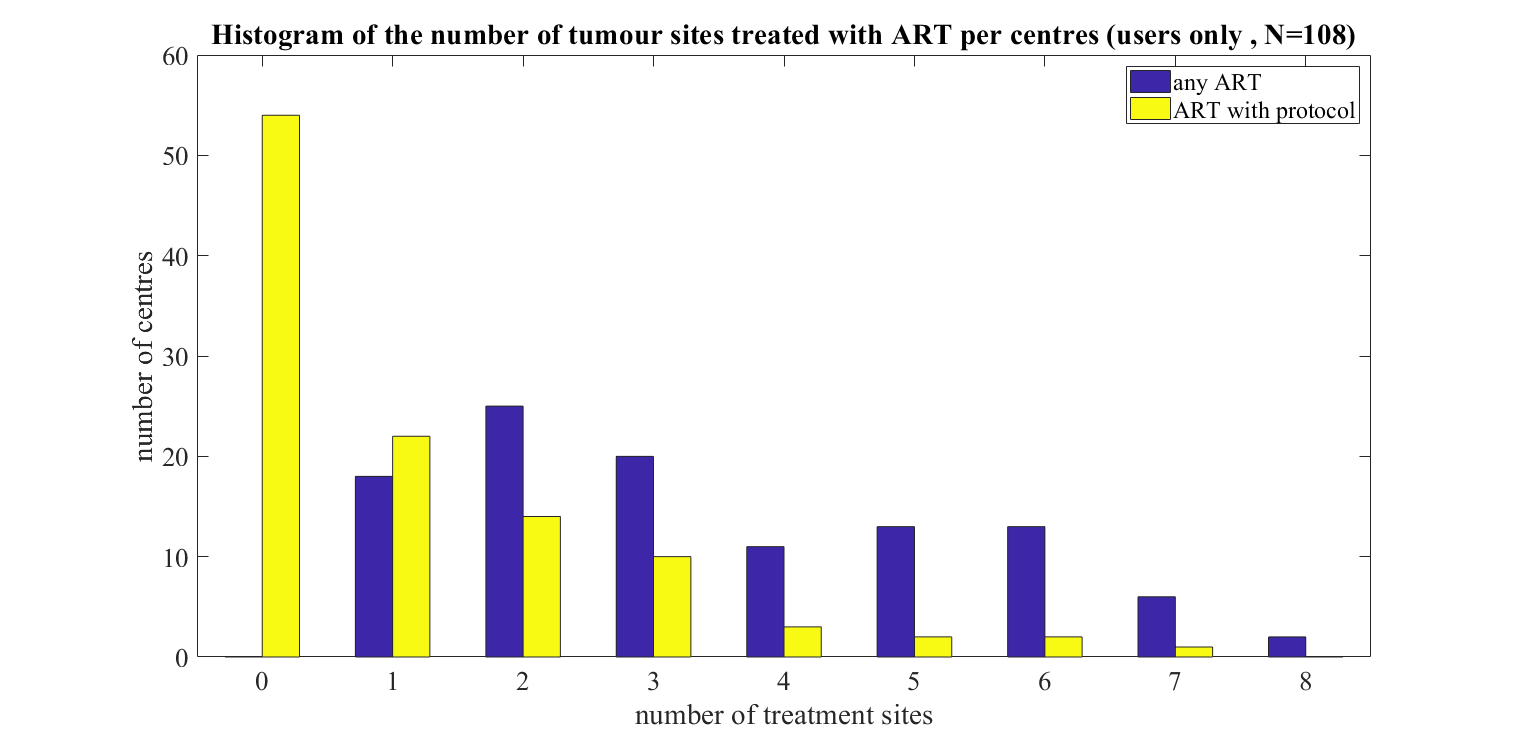

Supplement: Supplementary Fig. A.1 — Histogram of the number of tumour sites treated with ART per users. Half of the users do not use any type of protocolled ART (yellow bars). The median number of tumour sites treated with ART was 3. Half of these users used protocols. [file mmc1.zip › Supp1.png]

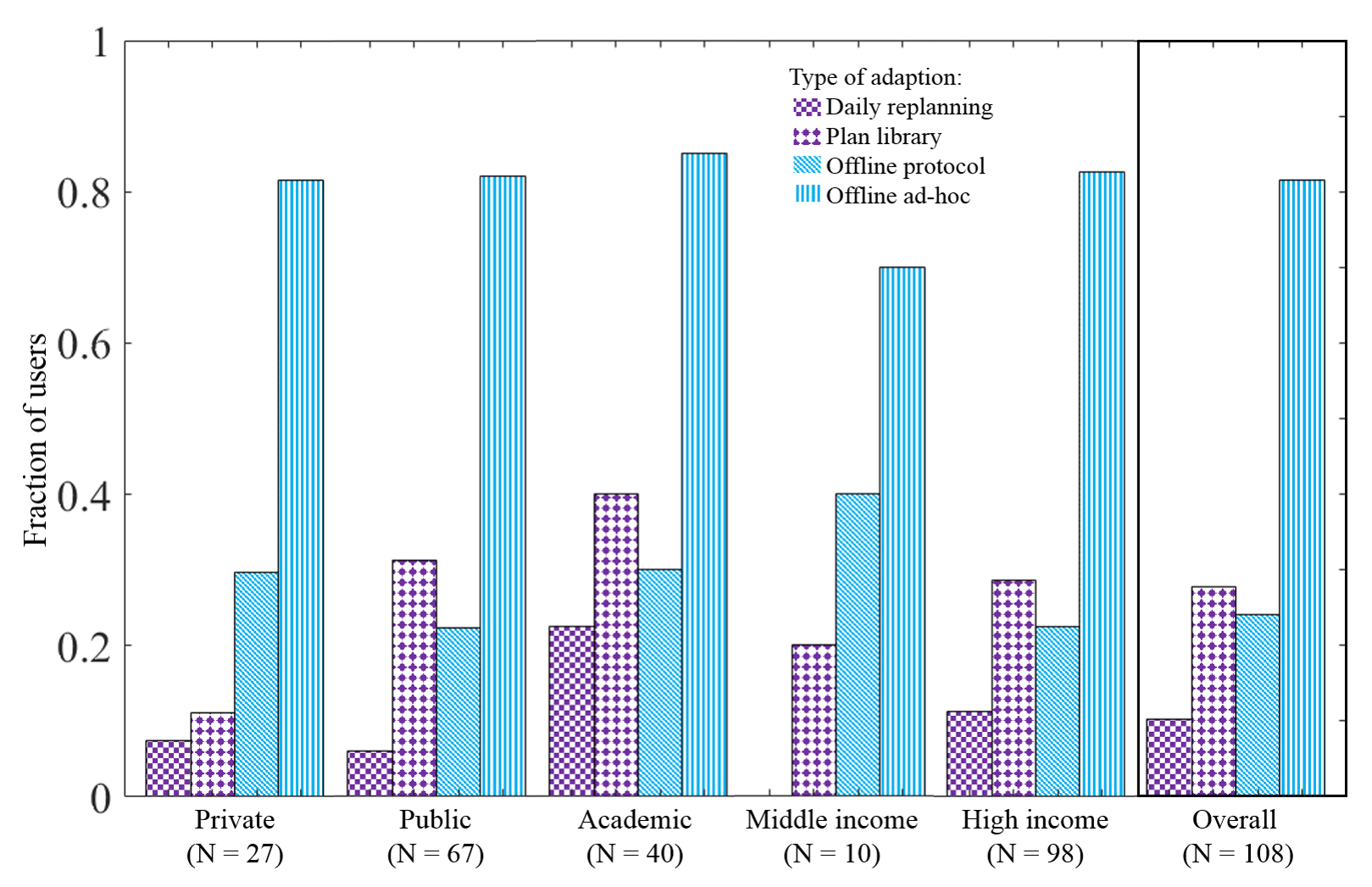

Supplement: Supplementary Fig. A.2 — Fractions of users that apply the defined types of ART. Data are presented for all users together (last column) and for subgroups defined by type of institution (private, public, academic) and income (middle or high-income). [file mmc2.zip › Supp2.png]

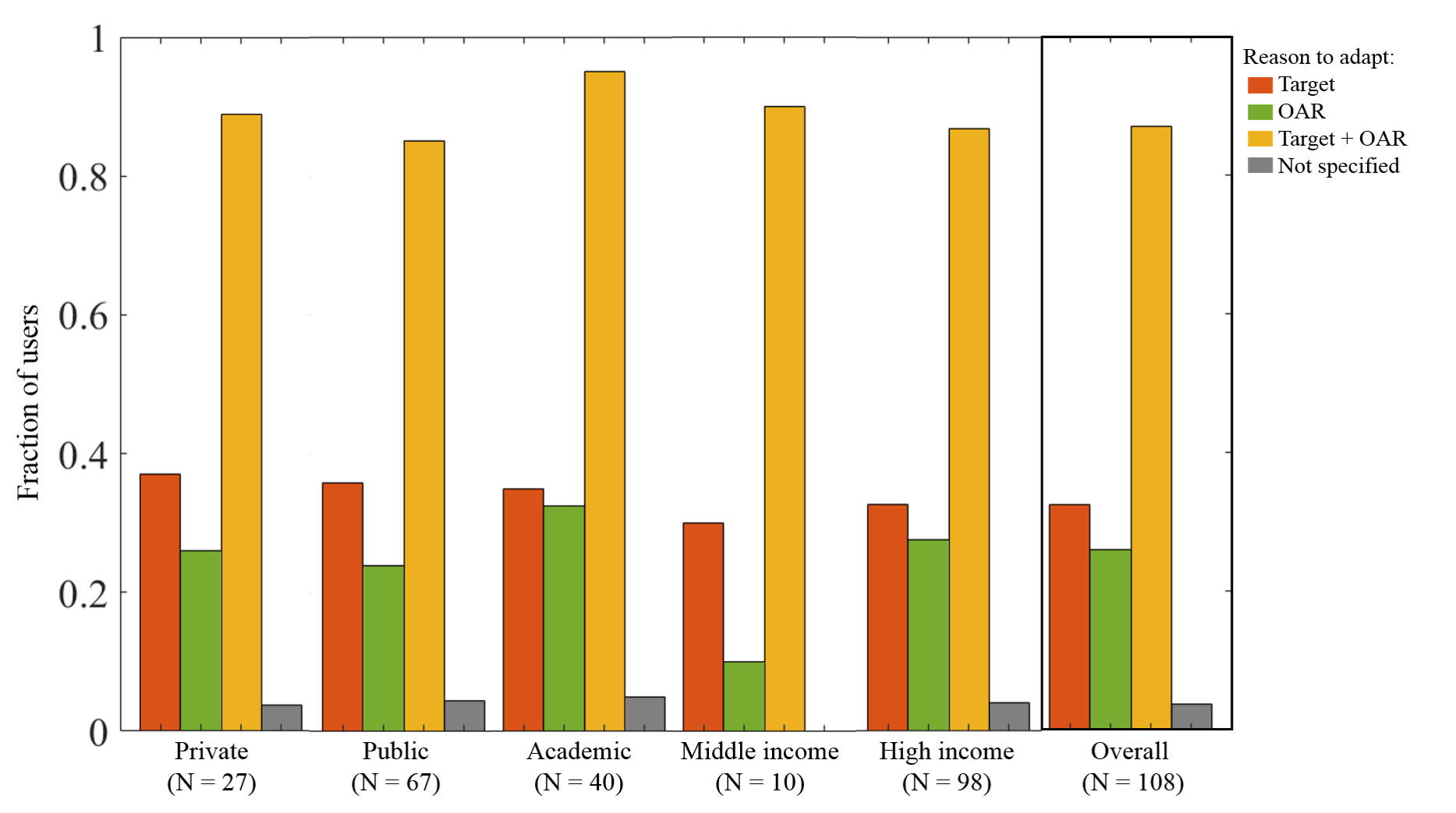

Supplement: Supplementary Fig. A.3 — Fractions of users that use ART to recover target dose (red), to improve OAR sparing (green), to improve both target coverage and OAR sparing (yellow) or for unknown reason (grey) per type of institution and economic status. The results for the overall group are shown to the right. [file mmc3.zip › Supp3.png]

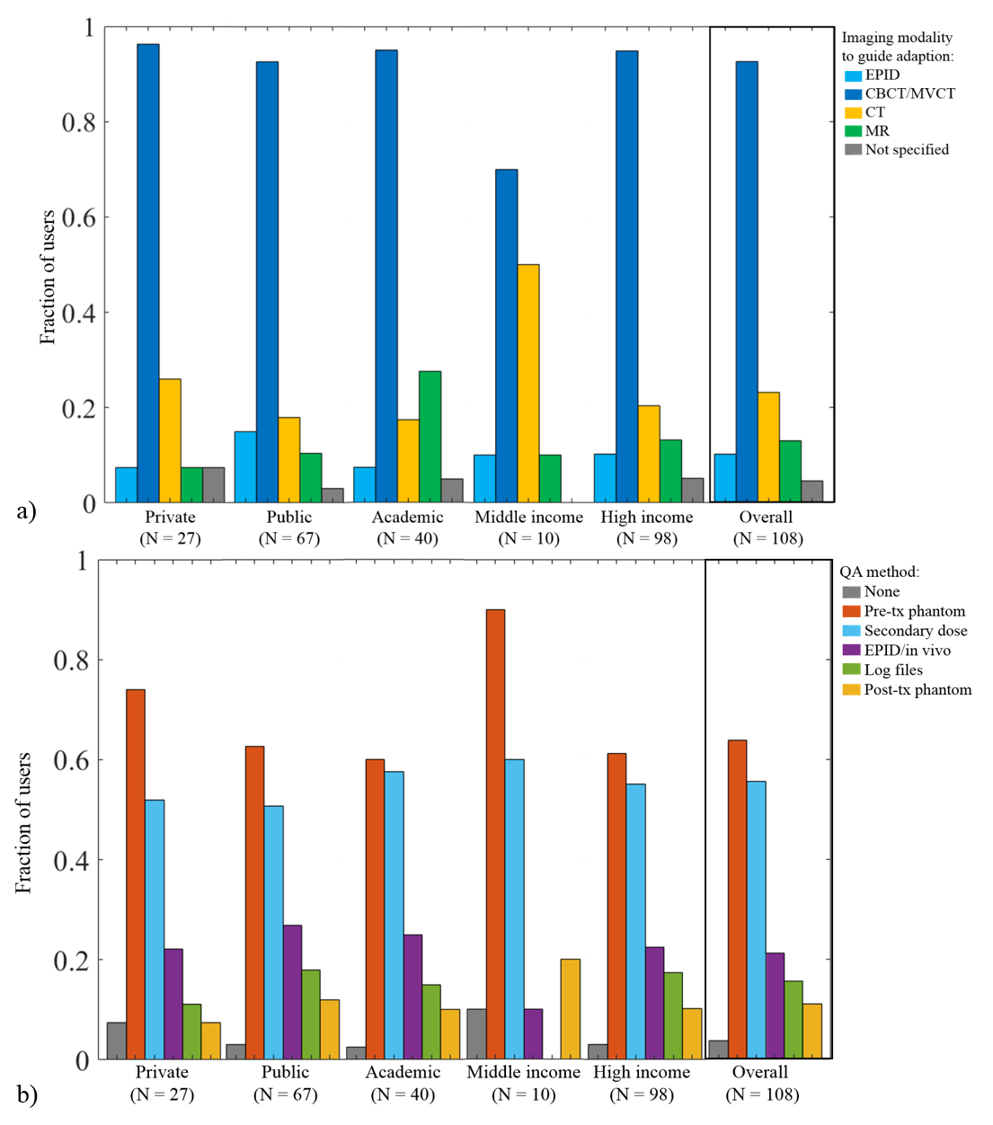

Supplement: Supplementary Fig. A.4 — a) Fractions of users that use given imaging modalities to guide the adaption (more than one response possible) per type of institution and economic status. b) Fractions of users that apply given QA methods (more than one response possible) per type of institution and economic status. [file mmc4.zip › Supp4.png]

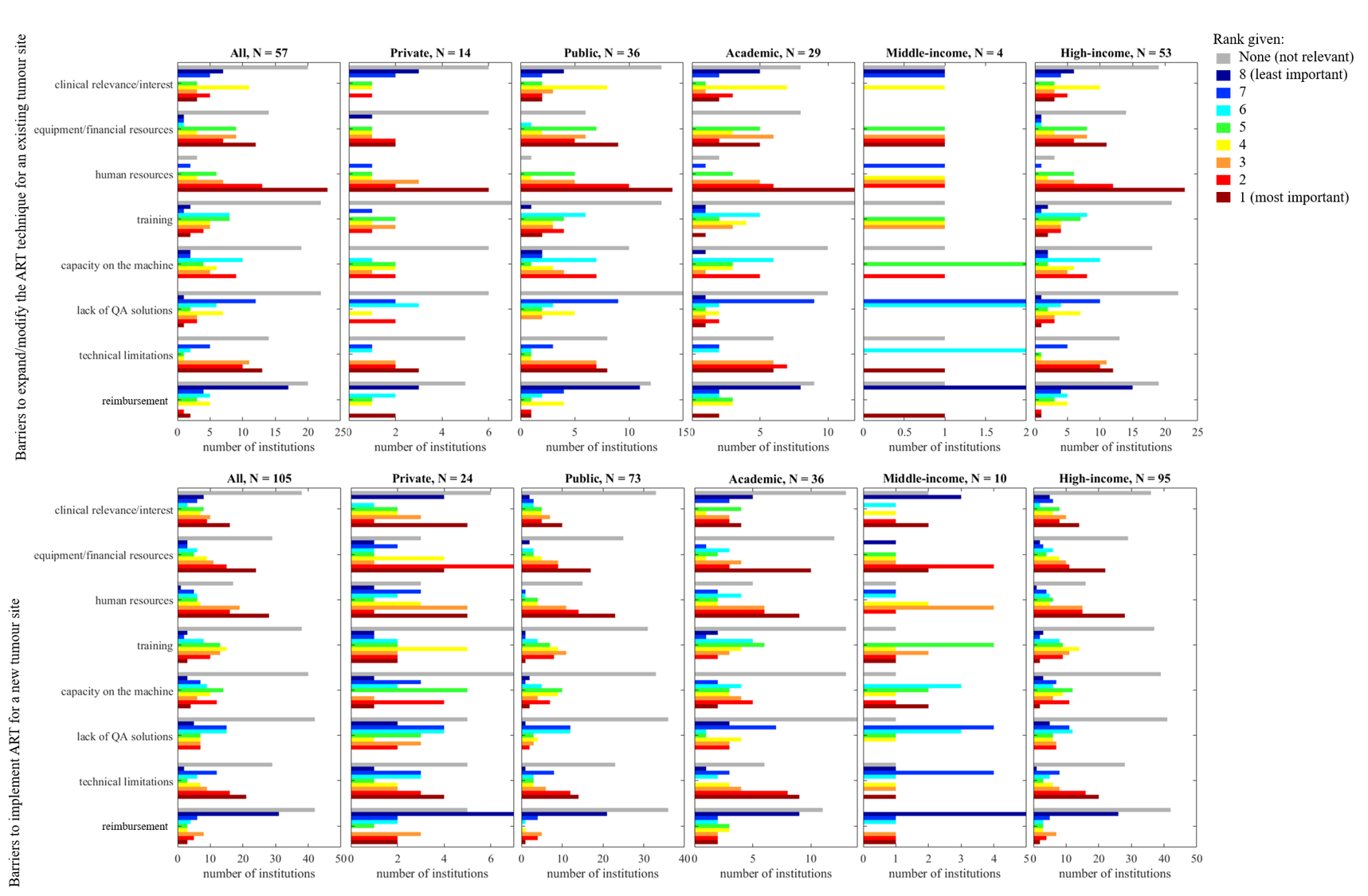

Supplement: Supplementary Fig. A.5 — Histogram of ranks given to the barriers to further use or new implementation of ART by type of institution and economic status. [file mmc5.zip › Supp5.png]
