# Supplementary material for: Patterns of practice for adaptive and real-time radiation therapy (POP-ART RT) part II: Offline and online plan adaption for interfractional changes
Source: Radiother Oncol. 2020 Dec;153:88–96. doi: 10.1016/j.radonc.2020.06.017 (PMC7758781; doi:10.1016/j.radonc.2020.06.017)
Supplement: Supplementary data: web-based questionnaire [file mmc6.pdf]

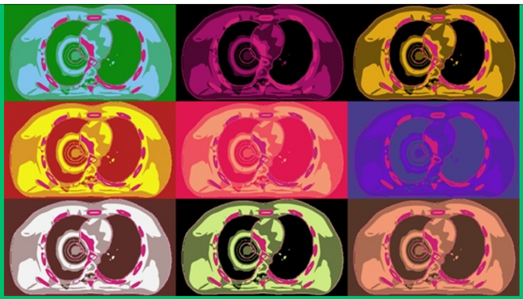

Pattern Of Practice for Adaptive and Real Time Radiation Therapy (POP-ART RT)

Introduction

Dear colleagues,

The present survey has been established by the participants of the 2nd ESTRO physics workshop from the track "realtime and adaptive management of anatomical variations" held in October 2018 in Málaga, Spain.

The aim of the Pattern Of Practice for Adaptive and Real Time Radiation Therapy (POP-ART RT) study is two-fold. Firstly, we would like to determine to which extent and how adaptive and real-time radiotherapy is being used in clinical practice. Secondly and more importantly, we would like to understand what the barriers to implementation or further use are.
For this reason, it is important that all institutions, including those that are not doing any form of adaptive or real-time radiotherapy, answer the survey. If your institution is not doing any form of real-time and adaptive radiotherapy, it will only take 5 minutes for you to answer!

The questionnaire is institution-specific and we encourage you to coordinate with your colleagues to provide one answer per institution. We understand that one person may not be able to answer all the questions. As long as you are using the same computer and browser (and do not erase cookies) you will be able to edit your responses until the survey closes. Please fill the survey online, however, a PDF of the questions is available upon request (please e-mail us) and can be used for internal discussion prior to online completion. We appreciate your effort and dedication to answer as completely as possible. The estimated time to complete the survey is 5-30 minutes depending on how extensively your institution is using adaptive and real-time radiation therapy.

The results of the survey will be disseminated in the form of scientific work. Ultimately, the results will allow us to identify the necessary actions to be taken by the vendors, the users and the society to implement adaptive and real-time radiation therapy more widely in clinical practice and increase confidence in the use of available technology.

We hope that you will find this project interesting and valuable. We thank you for your participation and we look forward to sharing the results of the POP-ART RT study with you and the RT community.

On behalf of the POP-ART RT workshop participants,
Jenny Bertholet, Gail Distefano, Ben Heijmen and Marianne Aznar
Contacts: Gail Distefano (gail.distefano@nhs.net) or Jenny Bertholet (jenny.bertholet@icr.ac.uk)

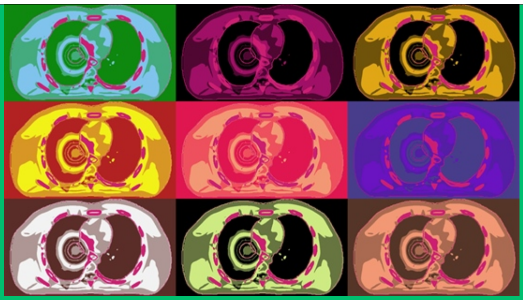

Pattern Of Practice for Adaptive and Real Time Radiation Therapy (POP-ART RT)

General information

1. What is the name of your institution ?

2. In which country are you situated ?

3. Your institution is ... (please tick all that apply)

- [ ] Private
- [ ] Public
- [ ] Academic

4. How many patients are treated with external beam radiotherapy per year in your institution (approximately)?

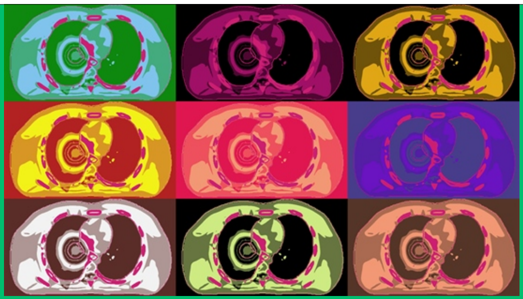

Intrafraction breathing motion management

1. Do you perform gating (free-breathing, breath-hold) or tracking for breathing motion management of any tumour site in your hospital?

NB: tracking means actively realigning the target and the beam (robotic tracking, gimbal tracking, MLC or couch tracking)

- ◯ Yes, continue to site-specific questions
- ◯ No, skip to plans and wishlist section

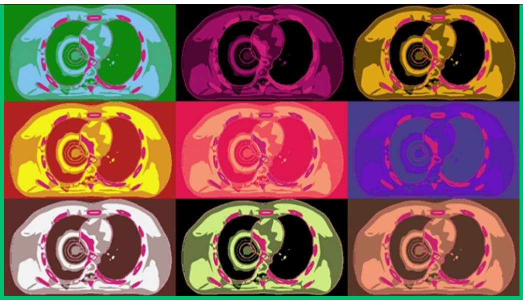

Pattern Of Practice for Adaptive and Real Time Radiation Therapy (POP-ART RT)

Current status : Intrafraction breathing motion management

1. For which tumour sites are you doing gating (free breathing, breath-hold) or tracking in your hospital for breathing motion management ?

- [ ] Breast
- [ ] Lung
- [ ] Liver
- [ ] Pancreas
- [ ] Other (please specify)

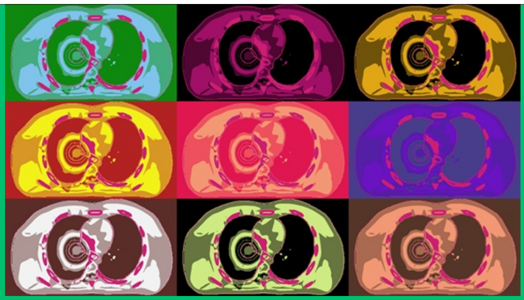

Pattern Of Practice for Adaptive and Real Time Radiation Therapy (POP-ART RT)

Current status: Intrafraction breathing motion management

Click **N/A** for the tumour sites for which you are **not** doing gating or tracking.
Note that breath-hold is considered as gating.

1. What percentage of patients within each tumour site are treated with gating/tracking?

	<25%	25-50%	50-75%	>75%	100%	N/A
Breast	○	○	○	○	○	○
Lung	○	○	○	○	○	○
Liver	○	○	○	○	○	○
Pancreas	○	○	○	○	○	○
Other 1 (if applicable)	○	○	○	○	○	○

2. What are the selection criteria to use gating/tracking for this cohort (e.g. only SBRT patients, based on performance status, based on motion amplitude measured pre-treatment,... please indicate if it is part of a research protocol)?

Breast	
Lung	
Liver	
Pancreas	
Other 1 (if applicable)	

3. On which machine(s) are you delivering the treatment with gating/tracking?

	Linac	Tomotherapy	Cyberknife	Vero	MR-linac	N/A
Breast	☐	☐	☐	☐	☐	☐
Lung	☐	☐	☐	☐	☐	☐
Liver	☐	☐	☐	☐	☐	☐
Pancreas	☐	☐	☐	☐	☐	☐
Other 1 (if applicable)	☐	☐	☐	☐	☐	☐

4. Which gating/tracking technique do you use?

	(Deep-) inspiration breath-hold	Expiration breath-hold	Free-breathing inspiration gating	Free-breathing expiration gating	Tracking	N/A
Breast	☐	☐	☐	☐	☐	☐
Lung	☐	☐	☐	☐	☐	☐
Liver	☐	☐	☐	☐	☐	☐
Pancreas	☐	☐	☐	☐	☐	☐
Other 1 (if applicable)	☐	☐	☐	☐	☐	☐

5. Do you have a separate training/coaching session?

	Yes	No	N/A
Breast	○	○	○
Lung	○	○	○
Liver	○	○	○
Pancreas	○	○	○
Other 1 (if applicable)	○	○	○

Breast – Yes (please specify how much time is dedicated to it in minutes)

Lung – Yes (please specify how much time is dedicated to it in minutes)

Liver – Yes (please specify how much time is dedicated to it in minutes)

Pancreas – Yes (please specify how much time is dedicated to it in minutes)

Other 1 (if applicable) – Yes (please specify how much time is dedicated to it in minutes)

6. Which gating/tracking signal are you using ? Here we mean the signal that is trigge the gating on/off or tracking feedback loop.

	External marker (e.g. RPM)	Surface monitoring (e.g. Vision RT)	Breathing volume (e.g. ABC)	Pressure belt (e.g. Anzai)	Implanted fiducial markers in kV images	Implanted fiducial markers in portal Imaging (EPID)	Markerless monitoring in kV/MV images	Implanted electromagnetic transponders (Calypso)	MR Imaging	I Sy V
Breast										
Lung										
Liver										
Pancreas										
Other 1 (if applicable)										

Other (please specify which and for which site)

7. Do you use audio and/or visual feedback to the patient?

	Audio	Visual	Audio and visual	None
Breast				
Lung				
Liver				
Pancreas				
Other 1 (if applicable)				

8. If applicable, do you acquire online verification images **during** beam-on to verify the accuracy of a surrogate signal?

	Yes, we look at them online	Yes, but we review them offline	No	N/A
Breast	◯	◯	◯	◯
Lung	◯	◯	◯	◯
Liver	◯	◯	◯	◯
Pancreas	◯	◯	◯	◯
Other 1 (if applicable)	◯	◯	◯	◯

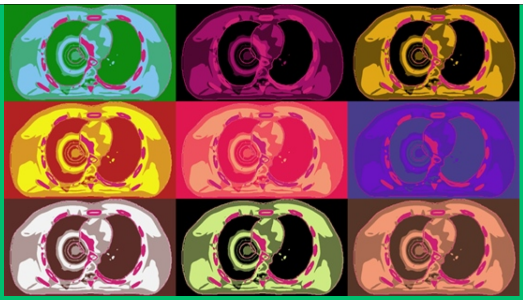

Pattern Of Practice for Adaptive and Real Time Radiation Therapy (POP-ART RT)

Plans and wishlists : intrafraction breathing motion management

1. Do you have plans to expand the use or change/improve your technique for gating or tracking for intrafraction breathing motion management for an existing tumour site in the next 2 years?

- ◯ Yes
- ◯ No
- ◯ Other (please specify)

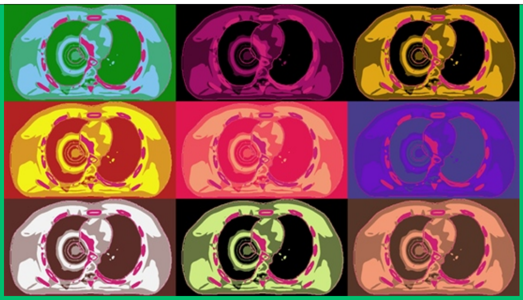

Pattern Of Practice for Adaptive and Real Time Radiation Therapy (POP-ART RT)

Barriers to wider use within one site

1. For which tumour site(s) do you wish to expand the use or change/improve your technique for gating/tracking in priority?

2. What are the main barriers/challenges to wider use? (rank in order of importance where 1 is the greatest challenge, leave the choices that are considered not relevant unmarked)

- Lack of clinical relevance/clinical interest
- Limited equipment/financial resources
- Limited human resources
- Lack of training
- Capacity of the machine
- Lack of QA solution
- Technical limitations (e.g. image quality, data connectivity, data flow...)
- Reimbursment

3. Any other main barrier not specified above?

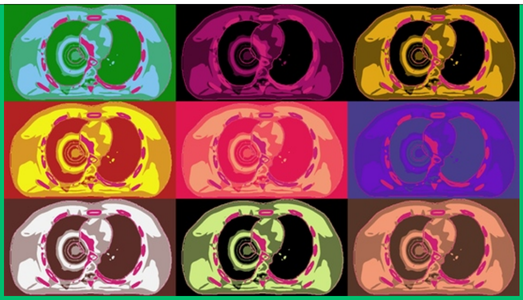

Plans and wishlists : intrafraction breathing motion management

1. Do you have wishes to implement the use of gating or tracking for intrafraction breathing motion management for a new tumour site?

- ◯ Yes, we want to implement gating or tracking for a new tumour site and we have plans for implementation in the next 2 years.
- ◯ Yes, but we have no plans to implement it.
- ◯ No, we have not wish to implement gating or tracking for a new tumour site.

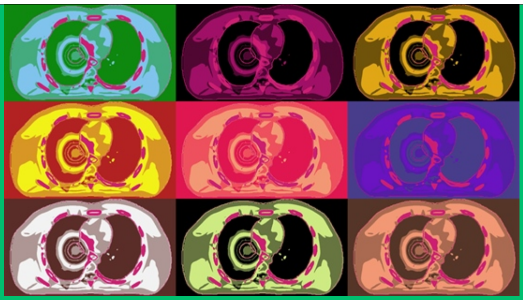

Pattern Of Practice for Adaptive and Real Time Radiation Therapy (POP-ART RT)

Barriers to new implementation of intrafraction breathing motion mitigation

1. For which tumour site(s) do you plan or would you like to do gating or tracking for intrafraction breathing motion mitigation in priority?

[ ]

2. What are the main barriers/challenges to implement gating/tracking for a new indication? (rank in order of importance where 1 is the greatest challenge, leave the choices that are considered not relevant unmarked)

- [ ] Lack of clinical relevance/clinical interest
- [ ] Limited equipment/financial resources
- [ ] Limited human resources
- [ ] Lack of training
- [ ] Capacity of the machine
- [ ] Lack of QA solution
- [ ] Technical limitations (e.g. image quality, data connectivity, data flow...)
- [ ] Reimbursment

3. Any other main barrier not specified above?

[ ]

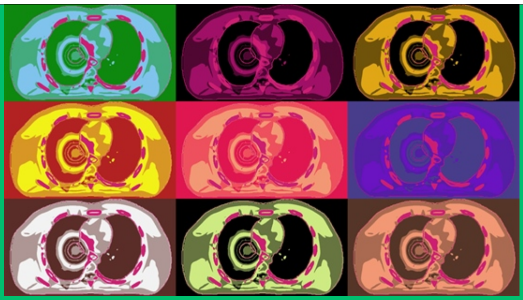

Pattern Of Practice for Adaptive and Real Time Radiation Therapy (POP-ART RT)

Adaptive radiotherapy for interfractional anatomical changes using multiple plans

By adaptive radiotherapy we mean: involving more than one plan per target per treatment course to cope with anatomical changes either by off-line replanning (e.g in case of tumor shrinkage), using a plan library approach or daily re-planning.

1. Are you doing adaptive radiotherapy for any tumour site in your hospital?

- ◯ Yes, continue to site-specific questions
- ◯ No, skip to plans and wishlist section

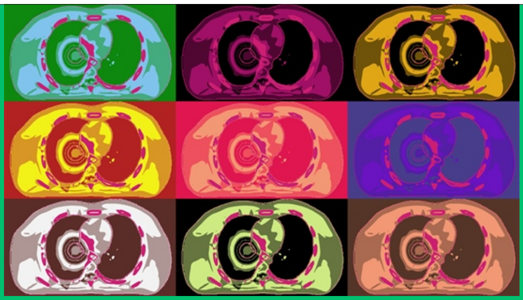

Pattern Of Practice for Adaptive and Real Time Radiation Therapy (POP-ART RT)

Current status : Adaptive radiotherapy for interfractional anatomical changes

1. For which tumour sites are you doing adaptive radiotherapy in your hospital ?

- [ ] Bladder
- [ ] Cervix
- [ ] Rectum
- [ ] Prostate
- [ ] Head and Neck
- [ ] Lung
- [ ] Other (please specify)

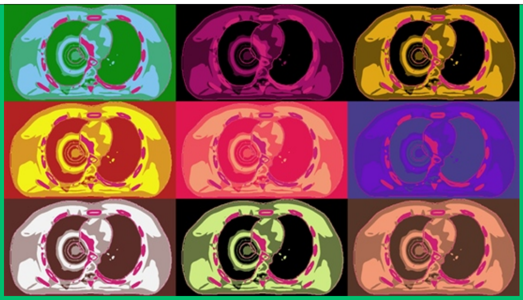

Pattern Of Practice for Adaptive and Real Time Radiation Therapy (POP-ART RT)

Current status: Adaptive radiotherapy

Click **N/A** for the tumour sites for which you do **not** do adaptive radiotherapy.

1. What type of adaptation are you using for this site

	Off-line ad-hoc replanning (e.g. in case of tumour shrinkage)	Off-line replanning with protocolled action level	Plan library approach	Daily replanning	N/A
Bladder	○	○	○	○	○
Cervix	○	○	○	○	○
Rectum	○	○	○	○	○
Prostate	○	○	○	○	○
Head and Neck	○	○	○	○	○
Lung	○	○	○	○	○
Other 1 (if applicable)	○	○	○	○	○

2. If off-line replanning (ad-hoc or protocolled): approximately what percentage of patients have more than 1 plan in total (i.e. one replan or more) ?

Bladder	
Cervix	
Rectum	
Prostate	
Head an Neck	
Lung	
Other 1	

3. Why are you doing adaptive radiotherapy (select all that apply)?

	Target dose considerations	OAR dose considerations	N/A
Bladder	☐	☐	☐
Cervix	☐	☐	☐
Rectum	☐	☐	☐
Prostate	☐	☐	☐
Head and Neck	☐	☐	☐
Lung	☐	☐	☐
Other 1 (if applicable)	☐	☐	☐

Other considerations (please specify which and for which site)

4. Which information is triggering the adaptation?

	CBCT/MVCT	MR	CT	EPID
Bladder	☐	☐	☐	☐
Cervix	☐	☐	☐	☐
Rectum	☐	☐	☐	☐
Prostate	☐	☐	☐	☐
Head and Neck	☐	☐	☐	☐
Lung	☐	☐	☐	☐
Other 1 (if applicable)	☐	☐	☐	☐

Other triggering (please specify which and for which site)

5. What kind of software is used for the adaptive procedure?

	In-house	Commercial (vendor specific or from another company)	Open source
Bladder	☐	☐	☐
Cervix	☐	☐	☐
Rectum	☐	☐	☐
Prostate	☐	☐	☐
Head and Neck	☐	☐	☐
Lung	☐	☐	☐
Other 1 (if applicable)	☐	☐	☐

6. If "in-house" at Q4: why not a commercial/open source solution?

	Too expensive	Not good enough	Does not offer the needed functionalities	Not commissioned	Lack of connectivity between software	No commercial or open source software available	N/A
Bladder	☐	☐	☐	☐	☐	☐	☐
Cervix	☐	☐	☐	☐	☐	☐	☐
Rectum	☐	☐	☐	☐	☐	☐	☐
Prostate	☐	☐	☐	☐	☐	☐	☐
Head and Neck	☐	☐	☐	☐	☐	☐	☐
Lung	☐	☐	☐	☐	☐	☐	☐
Other 1 (if applicable)	☐	☐	☐	☐	☐	☐	☐

Other (please specify)

7. What QA is performed on the new adapted plan(s)?

	None	Pre-treatment phantom measurements	Post-treatment phantom measurements	Secondary dose calculation	Log file analysis	EPID or in vivo dosimetry	N/A
Bladder	☐	☐	☐	☐	☐	☐	☐
Cervix	☐	☐	☐	☐	☐	☐	☐
Rectum	☐	☐	☐	☐	☐	☐	☐
Prostate	☐	☐	☐	☐	☐	☐	☐
Head and Neck	☐	☐	☐	☐	☐	☐	☐
Lung	☐	☐	☐	☐	☐	☐	☐
Other 1 (if applicable)	☐	☐	☐	☐	☐	☐	☐

Other (please specify which and which site)

8. Which tool do you use to manage and document plan adaptation?

	None	Record and verify	Spreadsheet (e.g. with Excel)	N/A
Bladder	○	○	○	○
Cervix	○	○	○	○
Rectum	○	○	○	○
Prostate	○	○	○	○
Head and Neck	○	○	○	○
Lung	○	○	○	○
Other 1 (if applicable)	○	○	○	○

Bladder – Other (please specify)

Cervix – Other (please specify)

Rectum – Other (please specify)

Prostate – Other (please specify)

Head and Neck – Other (please specify)

Lung – Other (please specify)

Other 1 (if applicable) – Other (please specify)

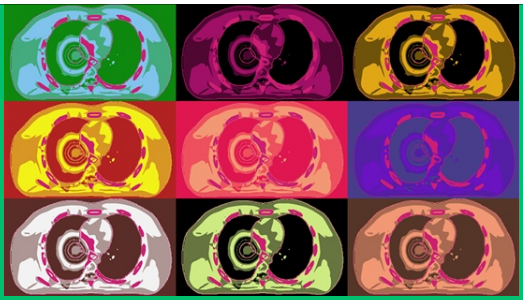

Pattern Of Practice for Adaptive and Real Time Radiation Therapy (POP-ART RT)

Plans and wishlists : adaptive radiotherapy

1. Do you have plans to expand the use or change/improve your technique for adaptive radiotherapy for an existing tumour site in the next 2 years?

- ◯ Yes
- ◯ No

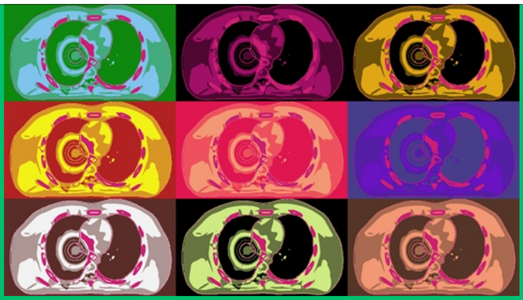

Pattern Of Practice for Adaptive and Real Time Radiation Therapy (POP-ART RT)

Barriers to wider use within one site

1. For which tumour site(s) do you wish to expand the use or change/improve your technique for adaptive radiotherapy in priority?

2. What are the main barriers/challenges to wider use? (rank in order of importance where 1 is the greatest challenge, leave the choices that are considered not relevant unmarked)

- Lack of clinical relevance/clinical interest
- Limited equipment/financial resources
- Limited human resources
- Lack of training
- Capacity of the machine
- Lack of QA solution
- Technical limitations (e.g. image quality, dose accumulation software, dose calculation speed, data connectivity, data flow...)
- Reimbursment

3. Any other main barrier not specified above?

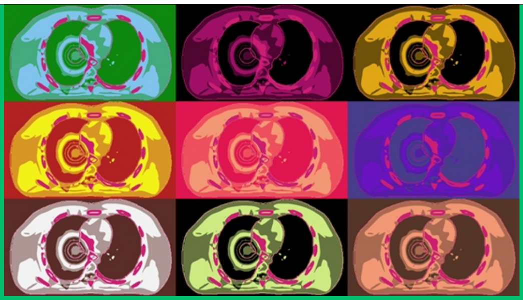

Plans and wishlists : adaptive radiotherapy

1. Do you have wishes to implement adaptive radiotherapy for a **new** tumour site?

- ◯ Yes, we want to implement adaptive radiotherapy for a new tumour site and we have plans for implementation in the next 2 years.
- ◯ Yes, but we have no plans to implement it.
- ◯ No, we have not wish to implement adaptive radiotherapy for a new tumour site.

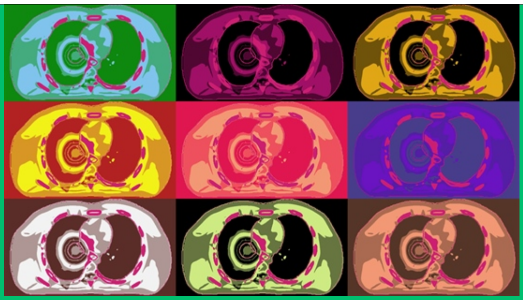

Pattern Of Practice for Adaptive and Real Time Radiation Therapy (POP-ART RT)

Barriers to new implementation of adaptive radiotherapy

1. For which tumour site(s) do you plan or would you like to implement adaptive radiotherapy in priority?

2. What are the main barriers/challenges to implement adaptive radiotherapy for a new indication? (rank in order of importance where 1 is the greatest challenge, leave the choices that are considered not relevant unmarked)

- Lack of clinical relevance/clinical interest
- Limited equipment/financial resources
- Limited human resources
- Lack of training
- Capacity of the machine
- Lack of QA solution
- Technical limitations (e.g. image quality, dose accumulation software, dose calculation speed, data connectivity, data flow...)
- Reimbursment

3. Any other main barrier not specified above?
