# Supplementary material for: Patterns of practice for adaptive and real-time radiation therapy (POP-ART RT) part II: Offline and online plan adaption for interfractional changes
Source: Radiother Oncol. 2020 Dec;153:88–96. doi: 10.1016/j.radonc.2020.06.017 (PMC7758781; doi:10.1016/j.radonc.2020.06.017)
Supplement: Supplementary Table A.1. [file mmc7.docx]

| Table A.1: Respondents characteristics. Number (percentage) | | | | | | | |
| --- | --- | --- | --- | --- | --- | --- | --- |
| Group | Overall N = 177 | Type of institution^1,4^ | | | Economic status^2,4^ | | |
|  |  | Private  N = 45 | Public  N = 114 | Academic  N = 52 | | Middle-income  N = 17 | High-income  N = 159 |
| Small volume^3^ | 53 (30%) | 22 (49%) | 29 (25%) | 4 (8%) | | 8 (47%) | 44 (28%) |
| Medium volume^3^ | 60 (34%) | 11 (24%) | 46 (40%) | 14 (27%) | | 5 (29%) | 55 (35%) |
| Large volume^3^ | 63 (36%) | 11 (24%) | 39 (34%) | 34 (65%) | | 3 (18%) | 60 (38%) |
| ^1^ Respondents could specify more than one type.  ^2^ Information unavailable for one respondent. There were no respondents from “low-income” countries. The “middle-income” group included centres from Egypt, Indonesia, India, the Philippines, Brazil, Ecuador, Mexico, Peru, Romania, Serbia, Russia and South Africa. The “high-income” group included centres from Austria, Belgium, Switzerland, Czech republic, Germany, Denmark, Estonia, Spain, Finland, France, Great Britain, Croatia, Hungary, Ireland, Italy, the Netherlands, Norway, Poland, Portugal, Sweden, Slovenia , USA, Canada, Australia, Israel, Hong Kong and the Arab Emirates  ^3^ Number of centres with small (<1000 patients/year) / medium (1000-2000 patients/year) / large (>2000 patients/year) patient volume (external beam RT only).  ^4^ One private centre from a middle-income country did not specify the patient volume. | | | | | | | |
