# Supplementary material for: Patterns of practice for adaptive and real-time radiation therapy (POP-ART RT) part II: Offline and online plan adaption for interfractional changes
Source: Radiother Oncol. 2020 Dec;153:88–96. doi: 10.1016/j.radonc.2020.06.017 (PMC7758781; doi:10.1016/j.radonc.2020.06.017)
Supplement: Supplementary Table A.2 [file mmc8.docx]

| Table A.2: Percentages of ART users; overall and according to type of institution and economic status. | | | | | | | | | | | | |
| --- | --- | --- | --- | --- | --- | --- | --- | --- | --- | --- | --- | --- |
| Group | Overall (N = 177) | | Type of institution^2^ | | | | | | Economic status^3^ | | | |
|  |  |  | Private  N = 45 | | Public  N = 114 | | Academic  N = 52 | | Middle  N = 17 | | High  N = 159 | |
| Type of adaption | **Online or offline protocol** | Offline  ad-hoc | **Online or offline protocol** | Offline ad-hoc | **Online or offline protocol** | Offline ad-hoc | **Online or offline protocol** | Offline ad-hoc | **Online or offline protocol** | Offline ad-hoc | **Online or offline protocol** | Offline ad-hoc |
| Bladder | **16%** | 11% | **7%** | 18% | **18%** | 7% | **25%** | 8% | **12%** | 17% | **16%** | 10% |
| Cervix | **13%** | 19% | **13%** | 20% | **11%** | 18% | **23%** | 21% | **12%** | 23% | **13%** | 19% |
| Rectum | **5%** | 13% | **7%** | 18% | **4%** | 11% | **12%** | 12% | **0** | 17% | **6%** | 13% |
| Prostate^1^ | **10%** | 18% | **11%** | 18% | **8%** | 18% | **19%** | 17% | **17%** | 12% | **9%** | 21% |
| Head and Neck | **10%** | 45% | **13%** | 44% | **7%** | 46% | **13%** | 58% | **17%** | 35% | **9%** | 47% |
| Lung | **8%** | 28% | **7%** | 29% | **7%** | 27% | **13%** | 33% | **17%** | 17% | **7%** | 29% |
| Breast ^1^ | **<1%** | 5% | **0** | 7% | **1%** | 4% | **2%** | 6% | **0** | 0 | **1%** | 5% |
| **Any site** | **31%** | **50%** | **24%** | 49% | **28%** | 48% | **48%** | 67% | **35%** | 41% | **30%** | 51% |
| ^1^Unspecified type of adaption for one user each.  ^2^ Respondents could specify more than one type.  ^3^ see table A.1. | | | | | | | | | | | | |
